# Protective Role of Genistein in Acute Liver Damage Induced by Carbon Tetrachloride

**DOI:** 10.1155/2007/36381

**Published:** 2007-04-10

**Authors:** Nalan Kuzu, Kerem Metin, Adile Ferda Dagli, Fatih Akdemir, Cemal Orhan, Mehmet Yalniz, Ibrahim Hanifi Ozercan, Kazim Sahin, Ibrahim Halil Bahcecioglu

**Affiliations:** ^1^Department of Internal Medicine, School of Medicine, Firat University, 23119 Elazig, Turkey; ^2^Department of Biochemistry, School of Medicine, Firat University, 23119 Elazig, Turkey; ^3^Department of Animal Nutrition, School of Veterinary, Firat University, 23119 Elazig, Turkey; ^4^Department of Animal Nutrition, School of Medicine, Firat University, 23119 Elazig, Turkey; ^5^Divison of Gastroenterology, School of Medicine, Firat University, 23119 Elazig, Turkey; ^6^Department of Pathology, School of Medicine, Firat University, 23119 Elazig, Turkey

## Abstract

*Aim*. In the present study, we investigated the protective effect of genistein in experimental acute liver damage induced by CCl_4_. *Method*. Forty rats were equally allocated to 5 groups. The first group was designated as the control group (group 1). The second group was injected with intraperitoneal CCl_4_ for 3 days (group 2). The third group was injected with subcutaneous 1 mg/kg genistein for 4 days starting one day before CCl_4_ injection. The fourth group was injected with intraperitoneal CCl_4_ for 7 days. The fifth group was injected with subcutaneous 1 mg/kg genistein for 8 days starting one day before CCl_4_ injection. Plasma and liver tissue malondialdehyde (MDA) and liver glutathione levels, as well as AST and ALT levels were studied. A histopathological examination was conducted. *Results*. Liver tissue MDA levels were found significantly lower in group 3, in comparison to group 2 (*P* < .05). Liver tissue MDA level in group 5 was significantly lower than that in group 4 (*P* < .001). Liver tissue glutathione levels were higher in group 5 and 3, relative to groups 4 and 2, respectively (*P* > .05 for each). Inflammation and focal necrosis decreased in group 3, in comparison to group 2 (*P* < .001 for each). Inflammation and focal necrosis in group 5 was lower than that in group 4 (*P* < .001). Actin expression decreased significantly in group 5, relative to group 4 (*P* < .05). *Conclusion*. Genistein has anti-inflammatory and antinecrotic effects on experimental liver damage caused by CCl_4_. Genistein reduces liver damage by preventing lipid peroxidation and strengthening antioxidant systems.

## 1. INTRODUCTION

Phytoestrogens are diphenolic molecules of plant origin, and resemble estradiol in structure and function. The major members of the phytoestrogen family are isoflavones, lignans, and coumestans. Of these, isoflavones are the most common and mostly studied phytoestrogens [1, 2]. Some epidemiological studies found phytoestrogens chemopreventive in cancer and coronary heart disease [3, 4].

Genistein is an isoflavone found primarily in the soy protein [5]. It has an estrogenic and antioxidant activity [6]. It was argued in previous studies that the beneficial effects of genistein were associated with its antioxidant effect [7, 8]. Administration of oral genistein was established to reduce lipid peroxidation in the liver and to increase total antioxidant capacity in hamsters [9]. Furthermore, genistein was found to inhibit tyrosine kinase, which accelerates tumor growth, and topoisomerase 1 and 2, and to prevent the formation of new capillaries, which is necessary for the growth of the tumor [1].

Carbon tetrachloride (CCl_4_) is a toxic agent used in experimental liver damage. It is metabolized by the mitochondrial monooxygenase (P450 2E1) system. During the metabolism, an unstable trichloromethyl (CCl_3_) free radical is formed, and rapidly converted to trichloromethyl peroxide (Cl_3_COO^−^) [10, 11]. These free radicals lead to the peroxidation of fatty acids found in the phospholipids making up the cell membranes. Lipid peroxide radicals, lipid hydroperoxides, and lipid breakdown products develop in this process, and each constitutes an active oxidizing agent. Consequently, cell membrane structures and intracellular organelle membrane structures are completely broken down. Structural damage spreads. Chronic administration results in fibrosis and cirrhosis [12]. Lipid peroxidation is important in liver damage associated with CCl_4_ [13, 14].

There are a limited number of studies about the protective effects of genistein on liver damage. In the present study, we aimed to explore the protective role of two different administration periods of genistein, which has antioxidant characteristics, in acute liver damage caused by CCl_4_.

## 2. MATERIAL AND METHOD

### 2.1. Experimental animals

The study included a total of 40 female Sprague-Dawley rats, weighing 200 to 260 g. The rats were equally allocated to 5 groups. They were kept in specially prepared cages in a room that had daylight for 12 hours. The study was carried out in accordance with ethical rules for standard experimental animal studies. The rats were fed with special rat feed supplied by Elazığ Feed Factory. Water was given through special dropper-tipped bottles placed specially in the cages.

### 2.2. Treatment regimen

**Figure 1:**
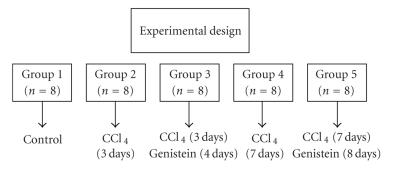
Schematic design of study.

Experimental design was shown in Figure 1. One of the five groups of rats was designated as the control group (group 1). The first group was injected with subcutaneous olive oil only. The second group was injected with intraperitoneal 0.15 ML/100 g CCl_4_ mixed with pure olive oil at a rate of 1/2, for 3 days (group 2). The third group was injected with subcutaneous 1 mg/kg genistein (Bonestein, Switzerland) for 4 days starting a day before CCl_4_ injection. CCl_4_ injection was continued for 3 days in the concerned group (group 3). The fourth group was injected with intraperitoneal 0.15 ML/100 g CCl_4_ mixed with pure olive oil at a rate of 1/2 for 7 days (group 4). The fifth group was injected with subcutaneous 1 mg/kg genistein in 0.2 ML serum physiologic for 8 days starting a day before CCl_4_ injection. CCl_4_ injection was continued for 7 days in the concerned group (group 5). Rats in groups 2 and 3 were decapitated on the fifth day, 24 hours after the treatment ended. Rats in groups 1, 4, and 5 were decapitated on the ninth day, 24 hours after the treatment was completed. Liver tissue and blood samples were collected for analysis, and stored at −20°C.

*Preparation of genistein*

Genistein was prepared by dissolving in 100 microliter DMSO (1.25%) and in the preparation of genistein (in the PGE) (98.75%) mixture and kept at +8°C [15].

Liver samples were collected from the rats for biochemical and histopathological examination. Tissue samples were collected accordingly for histopathological examination and tissue MDA and glutathione levels.

### 2.3. Plasma and tissue MDA measurements

Plasma MDA levels were determined according to thiobarbituric acid method modified by Yagi [16]. Results were expressed as nmol/ML. Liver tissue MDA levels were measured by Ohkawa method. Results were presented as nmol/g tissue [17].

### 2.4. Glutathione level

Determination of GSH was carried out as described by Sedlak and Lindsay [18]. Results were expressed as *μ*mol/mg tissue.

### 2.5. Biochemical parameters

The blood collected from the rats was centrifuged to separate plasma, which was kept at −20°C until analysis. Alanine aminotransferase (ALT), aspartate aminotransferase (AST), alkalene phosphatase (ALP), gamma glutamyl transpeptidase (*γ*-GT), urea, and kreatinin levels were measured with olympus kit (Olympus co; Japan) in Olympus AU 600 autoanalyzer.

### 2.6. Histopathological examination

Liver tissue samples were stored in 10% formalin solution to prepare paraffin blocks. Cross sections taken from the blocks were stained with hematoxylin eosin and masson trichrom. Histopathological examination was performed by a pathologist who specialized in this field. Percentage of steatotic cells was determined: grading was made as + up to 25%; ++ between 26 and 50%; +++ between 51 and 75%; ++++ >76%. Inflammatory cells were counted in randomly chosen 10 areas in x400 magnification, and mean cells by mm^2^ were determined by dividing the total by ten [19]. Necrosis foci were counted in randomly chosen 10 areas in x400 magnification, and mean necrotic foci by mm^2^ were established by dividing the total by ten [19, 20].

Cross-sections obtained from the paraffin blocks were stained with Masson Trichrom, and examined under x40, x100, x200, and x400 magnification. Fibrosis was staged between 0 and 4: no fibrosis: 0; fibrous portal extension: 1; bridging fibrosis: 3, cirrhosis: 4.

### 2.7. Immunohistochemical staining

In order to show hepatic stellate cell (HSC) activation, the liver tissue was stained immunohistochemically by *α*-SMA (actin smooth muscle neomakers, catalogue no. RB-910-R7). Presence of HSC reactive *α*-SMA in the liver was scored semiquantitatively [21]: grade 0: no or very rare staining; grade 1: staining of stellate cells in <30% of sinusoidal liver cells; grade 2: staining between 31 and 60%; grade 3: staining between 61 and 90%; grade 4: diffuse staining in more than 90% of sinusoidal liver cells.

### 2.8. Statistical evaluation

Data obtained from the study were presented as mean ± standard deviation. Kruskal Wallis one-way variance analysis was used in the comparison of parameters between groups, and Mann Whitney U test was employed in dual evaluations. SPSS 11.0 package software was used in the statistical evaluation. Level of statistical significance was set at *P* < .05.

## 3. RESULTS

One rat in group 5 died in the course of the study. Mean of the baseline body weight of the rats was comparable between the groups (*P* > .05).

### 3.1. Biochemical results

ALT and AST levels significantly increased in all groups, relative to the control group. ALT and AST levels were found higher in group 2, relative to group 1 (*P* < .001 for each), and significantly lower in group 3, relative to group 2 (*P* < .001, *P* < .05, resp.). ALT and AST levels in group 5 were lower than those in group 4 (*P* < .05 for each).

### 3.2. Lipid peroxidation and glutathione levels

Plasma MDA and liver tissue MDA levels in groups 2, 3, 4, and 5 increased relative to the levels in the control group. Liver glutathione levels in groups 2 and 4 decreased significantly in comparison to the control group. Liver tissue MDA level in group 3 (23.17 ± 2.82 nmol/g tissue) was significantly lower than that in group 2 (27.02 ± 2.54 nmol/g tissue) (*P* < .05). Plasma MDA level in group 3 was lower than that in group 3, but the difference was not significant (*P* > .05). Liver glutathione level was higher in group 3 (10.45 ± 1.3), relative to group 2 (8.72 ± 1.30) (*P* < .05).

Liver tissue MDA level in group 5 (33.78 ± 2.04 nmol/gr tissue) was significantly lower than that in group 4 (23.17 ± 2.82 nmol/gr tissue) (*P* < .001). Plasma MDA level was significantly lower in group 5 (3.41 ± 0.50 nmol/ML), in comparison to group 4 (4.14 ± 1.11 nmol/ML) (*P* < .001). Liver glutathione levels in group 5 (9.83 ± 0.88) were significantly higher than those in group 4 (7.97 ± 1.41) (*P* < .05).

Serum ALT, AST, plasma MDA, liver tissue MDA, and glutathione levels are presented in Table 1.

### 3.3. Histopathological results

Steatosis, inflammation and necrosis significantly increased in groups 2, 3, 4, and 5, relative to group 1 (*P* < .001 for each). Fibrosis and actin expression in groups 4 and 5 were higher than that in group 1 (*P* < .001). Inflammation and focal necrosis declined in group 3, in comparison to group 2 (*P* < .001 for each). There was not any significant difference between groups 2 and 3 in terms of steatosis, fibrosis, and actin expression (*P* > .05).

Inflammation and focal necrosis was found lower in group 5, relative to group 4 (*P* < .001). Actin expression in group 5 was less than that in group 4 (*P* < .05), but there was no significant difference between the groups in terms of fibrosis (*P* > .05).

**Figure 2:**
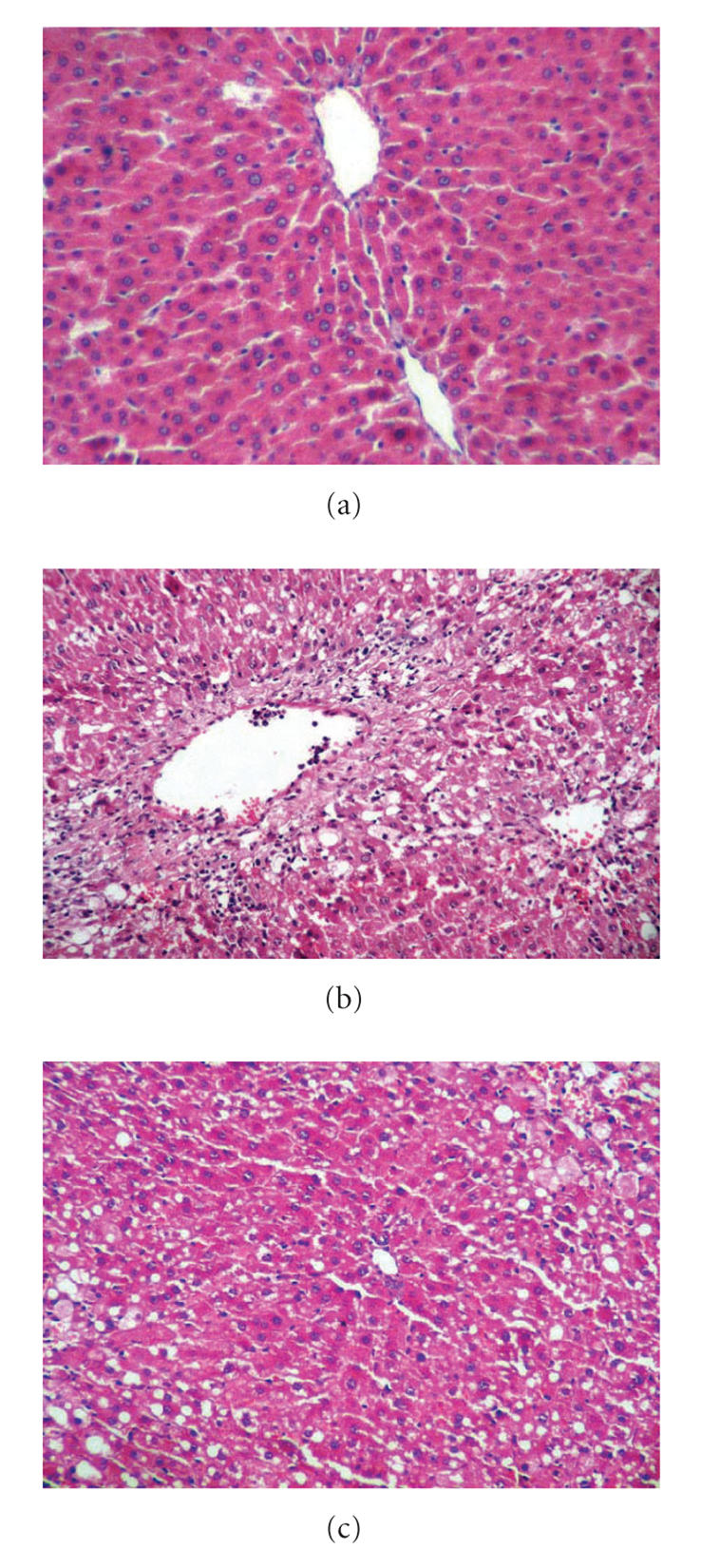
(a) Control (group 1): normal liver histology (H&E x200); (b) group 4: inflammation, necrosis was increased in group 4 (CCl_4_) (H&E x200); (c) group 5: inflammation and necrosis was decreased in group 5 (CCl_4_ +genistein): (H&E x200).

**Figure 3:**
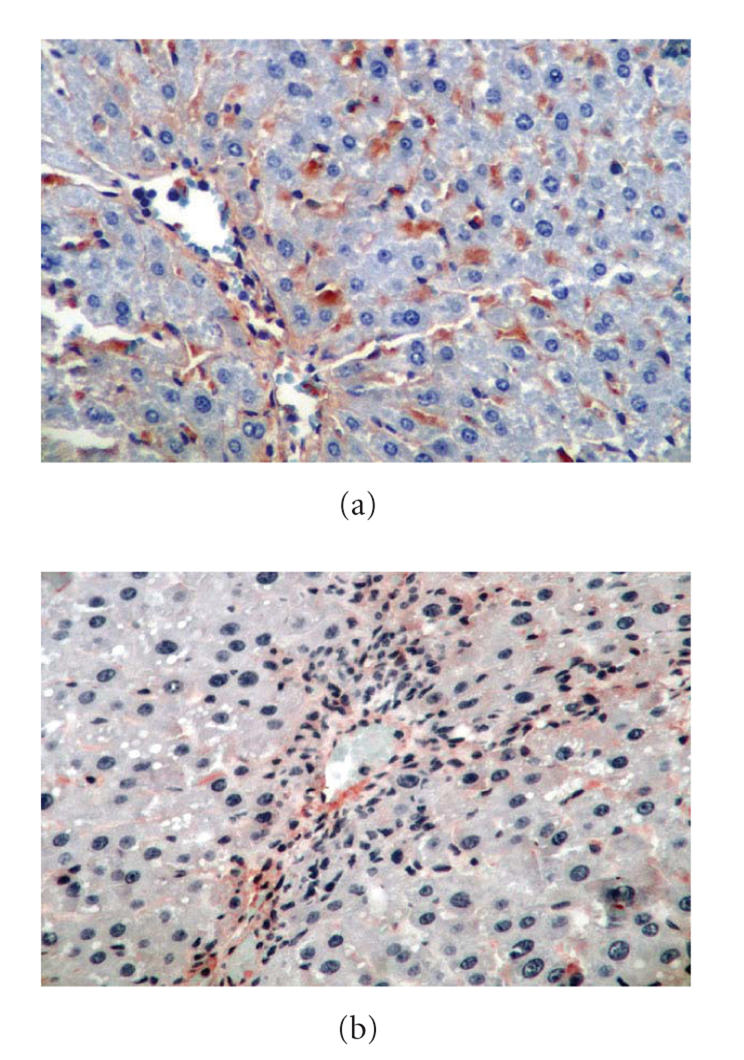
(a) Group 4: actine expression was increased in group 4 (x400); (b) group5: actine expression was decreased in group 5 (x400).

There was not any significant difference between groups with regard to steatosis (*P* > .05). Histopathological findings are presented in Table 2 and Figures 2 and 3.

## 4. DISCUSSION

Biotransformation of genistein mainly occurs in the liver and intestines. It is metabolized by cytochrome P450 system. It is converted into its monohydroxyl and dihydroxyl metabolites (7). Genistein has been in the spotlight of recent research since its discovery in 1987. There have been no side effects or toxicity reported with high doses of genistein in vivo [21]. In this study, administration of genistein for 3 and 7 days together with CCl_4_ brought about a significant decrease in liver tissue MDA levels. We also found a significant increase in glutathione levels.

In a recent study, genistein was compared with daidzein in liver damage induced by CCl_4_. In a study by Aneja et al. [22], genistein was administered orally, and CCl_4_ was injected on the forth and fifth days of the study. In our study, CCl_4_ was injected via intraperitoneal route on a daily basis for 3 and 7 days. Aneja et al. [22] did not perform a histopathological examination. They found that genistein elevated liver tissue glutathione levels, and reduced tissue lipid peroxidation levels.

Carbon tetrachloride is used to induce experimental liver damage. The toxic effect of CCl_4_ is due to the peroxidation of the membrane lipids by trichloromethyl (CCl_31/2_ or CCl_3_OO_1/2_) radicals [11, 12]. These radicals initiate lipid peroxidation chain reactions that start with taking hydrogen ions from polyunsaturated fatty acids (PUFA). Peroxidation of lipids containing polyunsaturated fatty acids, in particular, impairs the structure of biological membranes, causing severe cell damage. This has a significant role in the pathogenesis of many diseases [23]. Fang et al. [9] showed in their study that addition of 50 and 200 mg/kg genistein to the diet of hamsters with vitamin E deficiency inhibited oxidative stress in the liver tissue. Lipid peroxidation induced by dietary vitamin E deficiency was reduced by genistein administration. It was demonstrated that isoflavones elevated the cellular glutathione level in biological systems [24, 25]. Genistein can be effective in the detoxification of the toxic metabolites of CCl_4_ by restoring glutathione levels.

In this study, we established that genistein reduced inflammation and necrosis in both groups 3 and 5, relative to the groups injected with CCl_4_ only. This effect of genistein is probably associated with its inhibiting lipid peroxidation and preventing liver damage. However, it was also claimed that this anti-inflammatory effect was independent of the antioxidant effect. Anti-inflammatory effects of genistein were demonstrated in the study by Guo et al. [26] too. Genistein was shown to inhibit NO production from macrophages stimulated by LPS and IFN *γ*. In our study, levels of ALT and AST, which were the markers of liver necrosis and inflammatory activity, decreased by genistein administration.

Hepatic stellate cells (HSC) have an important role in liver fibrosis [27, 28]. Genistein brought about a significant decline in actin expression in group 5 only. HSC can be activated by many factors like cytokines, lipid peroxidation products, and so forth [27, 28]. Tyrosine kinase, one of the factors of HSC activation, has an important role in the proliferation and activation of HSC. Genistein can prevent activation and proliferation of HSC by inhibiting tyrosine kinase [29]. Genistein was shown to reduce type 1 procollagen expression in rat HSC-T6 cells and to inhibit the proliferation of HSC cells [30]. This effect of genistein was not marked at an early stage in our study. As time passes by, inhibition of actin expression acquires significance. Long-term procedures are needed to examine antifibrotic effects of genistein.

In conclusion, genistein can have a protective effect on liver damage from various aspects. It can prevent lipid peroxidation by interfering with the free radicals. It can also strengthen the antioxidant system. Furthermore, it can provide effective protection against liver damage through its anti-inflammatory effects.

## Figures and Tables

**Table 1:** Level of malondialdehyde (MDA) and gluathione, ALT, AST in groups.

Parameters	Control (group1; *n* = 8)	CCl_4_ 3 days (group 2; *n* = 8)	CCl_4_ +genistein (group 3; *n* = 8)	CCl_4_ 7 days (group 4; *n* = 8)	CCl_4_ +genistein (group 5; *n* = 7)

ALT (IU/L)	103.85 ± 55.58	723.62 ± 301.00	361.50 ± 39.89^(a)^	454.14 ± 56.66	379.14 ± 42.94^(b)^

AST (IU/L)	239.14 ± 82.05	867.00 ± 290.26	660.37 ± 84.87^(b)^	726.28 ± 137.40	561.00 ± 59.21

Plasma MDA (nmol/ML)	2.50 ± 0.54	4.14 ± 1, 11	3.20 ± 0.66	5.51 ± 0.71	3.4 ± 0.50^(c)^

Liver tissue MDA nmol/gr tissue	19.05 ± 1.43	27.02 ± 2.54	23.17 ± 2.82^(d)^	33.78 ± 2.04	27.20 ± 1.68^(c)^

Liver tissue gluthatione *μ*mol/mg tissue	10.48 ± 0.42	8.72 ± 1.30	10.45 ± 1.31^(e)^	7.97 ± 1.41	9.83 ± 0.88^(e)^

ALT: Alanine aminotrasferase, AST: Aspartate aminotransferase, MDA: Malondialdehyde.

^(a)^ *P* < .001; significantly lower in group 3 (CCl_4_ +genistein) than in group 2 (CCl_4_)

^(b)^ *P* < .05; significantly lower in group 3 (CCl_4_ +genistein) than in group 2 (CCl_4_) and in group 5 (CCl_4_ +genistein) than in group 4 (CCl_4_).

^(c)^ *P* < .001; significantly lower in group 5 (CCl_4_ +genistein) than in group 4 (CCl_4_).

^(d)^ *P* < .001; significantly lower in group 3 (CCl_4_ +genistein) than in group 2 (CCl_4_).

^(e)^ *P* < .05: significantly increased in group 3 (CCl_4_ +genistein) than in group 2 (CCl_4_); in group 5 (CCl_4_ +genistein) in than group 4 (CCl_4_).

**Table 2:** Results of histopathological findings.

Findings	Control (group 1; *n* = 8)	CCl_4_ 3 days (group 2; *n* = 8)	CCl_4_ +genistein (group 3; *n* = 8)	CCl_4_ 7 days (group 4; *n* = 8)	CCl_4_ +genistein (group 5; *n* = 7)

Steatosis (0–4)	—	1.13 + 0, 6	1.38 ± 0.5	3.00 ± 0.0	2.57 ± 0.5

Inflammation (cells/mm^2^)	1 ± 0.77	20.1 ± 3.9	12.8 ± 3.6^(a)^	19.29 ± 4.1	6.86 ± 2.4^(a)^

Necrosis (foci/mm^2^)	—	1.10. ± 0.2	0.7 ± 0.2^(a)^	1.87 ± 0.1	0.77 ± 0.2^(a)^

Fibrosis (0–4)	—	0.63 ± 0.6	0.88 ± 0.4	2.14 ± 0.9	1.57 ± 0.9

Actin (*α*-SMA) expression	—	1.38 ± 0.5	1.87 ± 0.2	2.86 ± 0.4	2.00 ± 0.5^(b)^

^(a)^ *P* < .001; significantly lower in group 3 (CCl_4_ +genistein) than in group 2 (CCl_4_) and in group 5 (CCl_4_ +genistein) than in group 4 (CCl_4_).

^(b)^ *P* < .05; significantly lower in group 5 (CCl_4_ +genistein) than in group 4 (CCl_4_).

## LIST OF ABBREVIATION

DMSO: Dimetyl suphoxide
PGE: Preparation of genistein
NO: nitric oxide
LPS: lipopolysaccaride.
